# Clinical significance and diagnostic approach for paediatric unilateral tonsillar enlargement: insights from a retrospective analysis

**DOI:** 10.1308/rcsann.2024.0113

**Published:** 2025-04-03

**Authors:** A Nelson, I Bujoreanu, J Gaskin

**Affiliations:** ^1^ Bristol Royal Hospital for Children, UK; ^2^ Grange University Hospital, UK; ^3^ Musgrove Park Hospital, UK

**Keywords:** Unilateral tonsillar enlargement, Paediatric, Lymphoma, Tonsillar asymmetry

## Abstract

**Background:**

One of the debated indications for paediatric tonsillectomy is unilateral tonsillar enlargement (UTE). The majority of UTE is innocuous in nature; however, concerns exist around a diagnosis of lymphoma, typically found in the presence of other symptoms.

**Methods:**

A retrospective case series analysis of all paediatric tonsillectomy specimens at Bristol Children’s Hospital between January 2006 and January 2023 was undertaken.

**Results:**

Four (1.3%) lymphoma diagnoses were identified from the 319 patients who underwent tonsillectomy for UTE. Three patients had localised disease and one patient had systemic infiltration of disease. All patients presented with other signs of malignancy including cervical lymphadenopathy (100%), alteration of appearance of tonsil including colour or visible lesion (75%), snoring (75%), dysphagia (50%), recurrent fever (25%) and weight loss (25%).

**Conclusions:**

We recommend active monitoring of asymptomatic isolated UTE. Diagnostic tonsillectomy should be performed in patients with UTE and cervical lymphadenopathy and/or constitutional symptoms and/or altered tonsillar appearance.

## Introduction

Tonsillectomy is one of the most common surgical procedures performed by paediatric otorhinolaryngologists.^1^ In 2016/2017, 37,000 tonsillectomies were performed in the UK, with estimated costs to the NHS totalling £42 million.^2^ Indications for tonsillectomy include recurrent tonsillitis, previous peritonsillar abscess, obstructive sleep apnoea and suspicion of malignancy.^3^ Lymphoma is the most common malignancy of the head and neck in childhood and accounts for 12% of all cancers in under 15-year-olds.^4,5^ Extranodal involvement is most often seen in non-Hodgkin lymphoma (NHL), with tonsillar involvement being most frequent and seen in up to 20% of paediatric cases.^6^

One of the debated indications for tonsillectomy is unilateral tonsillar enlargement (UTE), which is present in 1.7% of children aged 4–17 years old.^4^ The majority of UTE is innocuous in nature, including benign hyperplasia, inflammation, infection and anatomical variation.^7^ There have been multiple studies reporting no objective difference in tonsillar size on histopathological examination, and the appearance of UTE may be attributed to variations in the depth of the tonsillar fossa or anterior tonsillar pillar asymmetry, rather than true difference in tonsillar size.^7,8^

The most common presentation of tonsillar lymphoma is UTE, which is present in 73.2% of tonsillar lymphoma cases. However, isolated tonsillar lymphoma is rare and represents less than 1% of cases of head and neck cancer.^9^ The suspicion of tonsillar lymphoma is significantly increased when UTE is found in the presence of other sinister signs including rapid tonsillar enlargement, lymphadenopathy, suspicious appearance of tonsillar mucosa and constitutional symptoms including weight loss, anorexia and fatigue.^8,9^

Despite the low prevalence of occult malignancy in isolated UTE (0.35%), it had largely become common practice for otorhinolaryngologists to perform diagnostic tonsillectomy in the presence of UTE, even when no other sinister features exist.^4,10^ Ongoing controversy exists regarding the clinical justification of diagnostic tonsillectomy in the context of isolated UTE, given the low yield, high costs and paediatric complications, alongside the ongoing funding constraints faced within the NHS.^8,11,12^

## Methods

A retrospective case series analysis was performed of all paediatric tonsillectomy specimens, at a single-centre tertiary paediatric hospital over a 17-year period. All operations were performed at Bristol Children’s Hospital between January 2006 and January 2023. Cases were included where the primary indication for tonsillectomy was UTE. Common features were assessed to ascertain whether those with malignancy could be identified more easily from those with benign asymmetry. Cases of known malignancy undergoing tonsil biopsy/tonsillectomy due to observed tonsillar or to biopsy radiological areas of concern were excluded as the primary indication was not UTE. Anonymised data collection included patient demographic details, clinical history (including history of constitutional symptoms or lymphadenopathy to ascertain suspicion of malignancy) and histopathological results. Departmental approval was sought to facilitate collection of data; no formal ethical approval was required due to the nature of data collection.

## Results

Over the 17-year period, a total of 319 patients underwent tonsillectomy for UTE and had tonsillectomy specimens sent for histopathology. Of the 319 patients, 163 were female (51.1%) and 156 were male (48.9%). Patient age ranged from 7 months to 17 years and 3 months; the mean age of patients was 7.84 years. Out of the 319 patients undergoing tonsillectomy for UTE, there were four (1.3%) malignancies identified, all of which were lymphoma diagnoses. There were three male patients and one female patient with lymphoma.

One patient presented with UTE, and the initial histology report suggested low-grade follicular lymphoma; however, on repeat histopathological analysis this case was reported as typical follicular hyperplasia. There were two additional patients who were identified with malignancy; however, on further analysis of the patients’ history, both patients already had known lymphoma diagnoses and the tonsillectomies were not performed on the indication of UTE and therefore were excluded from our study. The presenting symptoms in these patients were not related to altered tonsillar size/symmetry.

Of the four patients diagnosed with lymphoma, their ages ranged from 4 to 13 years. All of these patients presented with other signs of malignancy in addition to UTE, including cervical lymphadenopathy (100%), alteration of appearance of tonsil including colour or visible lesion (75%), snoring (50%), dysphagia (50%), recurrent fever (25%) and weight loss (25%) (Table 1). There was a family history of malignancy in three out of four patients of varied aetiologies. Interestingly, lactate dehydrogenase (LDH) levels were raised in three patients. We were unable to obtain data on LDH levels for one patient (Patient 4). Of the three blood films that were performed, all samples appeared normal on examination.

**Table 1:** Presentation, history and blood results of confirmed malignancy cases in patients presenting with unilateral tonsillar enlargement

Patient^a^	1	2	3	4
Age at diagnosis (years)	10	4	13	4
Presentation date	October 2018	November 2014	May 2013	February 2005
Histology date	2 October 2018	11 November 2014	24 May 2013	11 February 2005
Enlarged tonsil side	Right	Left	Left	Right
Procedure/biopsy date	Right tonsillectomy+excision right lymph node	Left tonsillectomy	Bilateral tonsillectomy	Bilateral tonsillectomy
Alteration of appearance	✓	✓	✓	⨯
Cervical lymphadenopathy	✓	✓	✓	✓
Dysphagia	✓	⨯	✓	⨯
Snoring	✓	✓	⨯	⨯
Recurrent fever	✓	⨯	⨯	⨯
Weight loss	✓	⨯	⨯	⨯
Immunosuppression	⨯	⨯	⨯	⨯
Previous radiotherapy	⨯	⨯	⨯	⨯
Any history of malignancy	⨯	⨯	⨯	⨯

^a^Represents missing data/data not collected during admission, ✓ yes, ⨯ no

All confirmed malignancies were lymphomatous in nature. There were two patients with Burkitt’s lymphoma, one patient with B-cell NHL and one patient with T-cell NHL (Table 2). Macroscopic appearances of histology specimens are outlined in Table 2. Cytogenetic testing on specimens demonstrated increased uptake on CD20 (75%) and CD79a, CD10, CD19 and Ki67 receptors in 50% of patients.

**Table 2:** Confirmed malignancy histological findings of patients presenting with unilateral tonsillar enlargement

Patient	1	2	3	4
Histology	Burkitt's lymphoma (stage II) (high grade)	Burkitt's lymphoma (high grade)	High grade (diffuse large) B cell NHL	T-cell NHL (R tonsil, R Cervical LN) Stage II
Tonsil size	R, 42×30×25mm	L, 40×30×20mm	L, 10g 40×20×18mm; R, 3g 27×15×10mm	R, 7g 32mm greatest dimension in section; L, 3g Maximum diameter 28mm.
Macroscopic appearances	R, firm gelatinous 42×30×25mm lymph nodule- gelatinous pink 33×22×18mm	L (fresh), white polypoid 8.2g 40×30×15mm L (fixed), 1.5g 25×12×12mm	L, On section fleshy, creamy, abnormal, lymphomatous; R, normal appearance	R, solid tan coloured in section; L, fleshy solid structure

R = Right; L = Left; NHL = Non-Hodgkins Lymphoma.

Diagnostic imaging results are presented in Table 3. Diagnostic and staging imaging was variable between patients as was the extent of disease. Three patients had localised disease, whereas one patient had systemic infiltration with oropharyngeal and abdominal disease (Table 3).

**Table 3:** Imaging of confirmed malignancy cases in patients presenting with UTE

Patient	1	2	3	4
CXR	Normal	Normal	Normal	Normal
				
USS	3.6cm×1.7cm right level 2 node-Ovoid, lost normal fatty hilum, hypervascular appearance	Bilateral renal infiltration with 3cm renal parenchymal nodules	Normal	Not performed
CT	Not performed	Bulky oropharynx disease, with partial oropharyngeal obstruction Left palatine tonsillar mass 3.5×3.5×3.5cm. Mass merged with pterygoid muscle with significant airway narrowing	Not performed	Right-sided moderate cervical lymphadenopathy, few small left-sided nodes Minor asymmetry of pharyngeal wall, slightly bulky on right vs left
MRI	Localised disease (cervical)	Oropharyngeal and abdominal disease	Normal	Not performed
Diagnosis	Burkitt’s lymphoma (stage II) (high grade)	Burkitt’s lymphoma (high grade)	High-grade (diffuse large) B-cell NHL	T-cell NHL (Right tonsil, Right Cervical LN) Stage II

NHL = Non-Hodgkin Lymphoma; CXR = Chest X Ray; USS = Ultrasound Scan; CT = Computed Tomography; MRI = Magnetic Resonance Imaging.

There were five (1.6%) patients with missing histology data, leaving 308 (96.6%) patients with benign histology results. The most common benign histological diagnosis was hyperplasia, accounting for 130 (42.2%) patients. Second to this were reactive/inflammatory changes, with 80 (25.6%) patients affected. Other common results included normal histology (13.3%), tonsillitis (3.7%) and actinomyces (3.4%).

## Discussion

This study identified a low incidence of malignancy associated with UTE (1.3%); there were no cases of malignancy identified in patients presenting solely with asymptomatic UTE. This is consistent with previous findings by Sunkaraneni *et al*, who reported 0% malignancy rates in isolated UTE.^10,13^ Notably, clinically abnormal tonsil features with significant asymmetry and associated cervical lymphadenopathy were present in all positive malignancy cases. This is also supported by a previous systematic review by Guimaraes *et al*, which reported a significant rate of malignancy in the presence of other sinister features.^8,10,14^ Other studies have reported low incidence of malignancy in isolated UTE, and concluded UTE in the absence of other symptoms is most likely to represent a benign pathology.^13,14^

Given the low incidence of malignancy in isolated UTE, careful consideration should be taken when proposing ongoing management of these patients. Further diagnostic tests or surgical management should be considered if history and examination of patients raises concern for malignancy. This includes rapid tonsillar enlargement, altered appearance of tonsillar mucosa, lymphadenopathy and organomegaly. Other concerns that may prompt diagnostic tonsillectomy include previous history of cancer, immunodeficiency or unresolving illness.^13,14^ It may be more appropriate to adopt regular observation and safety netting of paediatric patients presenting with isolated UTE, thus avoiding unnecessary risks posed by diagnostic tonsillectomy.

Diagnostic tonsillectomy for UTE is associated with its own perioperative risks and morbidity relating to postoperative haemorrhage, which has been reported in up to 12.1% of cases in a recent UK multicentre study.^15^ Unfortunately, we were unable to obtain data relating to postoperative morbidity in our cohort, as many of the case files from the study were archived following the introduction of online patient notes in 2017.^12,16^

Assessing the size of tonsils in clinical practice often poses challenges, due primarily to the inherent subjectivity and variability in interpretation. The Brodsky scale – a tool commonly used for tonsillar evaluation – can yield varying assessments when applied by different clinicians.^17^ Moreover, studies have suggested that the observed asymmetry in tonsil size is frequently attributed to differences in the tonsillar fossae rather than differences in tonsillar volume. Clinical tonsillar asymmetry is often apparent rather than objective. This further complicates tonsillar assessment and accuracy of tonsillar size, implying that apparent asymmetry may not necessarily signify pathology.^18,19^

Although UTE is the most common presenting symptom in tonsillar lymphoma, it will often represent a normal variant in the paediatric population. Previous studies have suggested UTE prevalence of 1.7% in healthy paediatric patients, with 210,000 children in the UK estimated to have UTE.^20^ This highlights the underlying importance of careful clinical evaluation of patients presenting with UTE, paying close attention to both history and clinical examination to identify red flags for malignancy that may warrant further investigation. In the context of the current study, all malignant tonsils exhibited grossly clinically abnormal features at preoperative examination, indicating further workup and diagnostic tonsillectomy.^11^ In those presenting with clinically abnormal features, we would recommend tonsillectomy to aid diagnosis as there is risk of an inadequate sample or false negative results in tonsillar biopsy.^21^

Current screening tests in our department for a child presenting with UTE include blood test including full blood count (FBC), liver function, renal function, C-reactive protein (CRP) and LDH. Additionally, a plain radiograph is used to assess mediastinal mass. A consensus on unified screening tests for children presenting with UTE has not yet been reached nationally, so our screening test profile is based largely on expert opinions within the trust. FBC in NHL may reveal anaemia, thrombocytopenia, leukopenia, pancytopenia, lymphocytosis or thrombocytosis, which can be due to extensive bone marrow infiltration, splenic involvement or blood loss.^22^ CRP can be used to rule out acute tonsillitis in combination with clinical assessment and is also a valuable prognostic marker in NHL.^23^

The current screening approach is limited as many investigations are nonspecific and LDH is raised in only 40% of cases, highlighting the importance of clinical assessment and history taking.^24^ However, a high LDH can represent high tumour burden and/or extensive liver or kidney infiltration; hence, the rationale for LDH, renal function and LFT.^22,25^ Furthermore, children rarely present with findings on chest x-ray. Recently, end-oral ultrasound to has emerged as a novel assessment tool of tonsillar tissue in children.^26^ In the future, integrating ultrasound in the assessment of UTE in children may enhance diagnostic accuracy, and complement the other screening tests performed to investigate possible underlying malignancy. In isolated UTE, reassuring ultrasound scan (USS) investigating for cervical lymphadenopathy and the absence of abnormal investigations including FBC, CRP and LDH, may advocate for observational management of children rather than diagnostic tonsillectomy.

## Conclusion

The presence of UTE in paediatric patients is common, and may be attributed to variations in assessment and tonsillar fossa depth, rather than from true UTE. Although there is an association with tonsillar malignancy, it is rare, accounting for only 1.3% of patients in our cohort. When malignancy does occur, it is typically characterised by grossly abnormal tonsillar appearance, cervical lymphadenopathy and/or concerning systemic signs and symptoms. Thorough history taking, clinical examination and direct visualisation by an otorhinolaryngologist are crucial, underscoring their essentiality for evaluating patients with UTE in the outpatient setting.

We suggest adopting an observational approach to isolated UTE in paediatric patients unless other concerning features for malignancy exist. Persistent UTE in the absence of other clinical signs could be further investigated with cervical USS to investigate for cervical lymphadenopathy and blood tests to include FBC, CRP and LDH. Any abnormalities in these investigations would warrant diagnostic tonsillectomy. Those presenting with any concerning signs and symptoms should undergo diagnostic tonsillectomy to investigate for possible malignancy.

## Data sharing and availability

The datasets used during the current study are available from the corresponding author on reasonable request.

## Authorship contributions

AN is the main contributor to data analysis, manuscript writing and editing, and agrees to be accountable for the work. IB contributed to data collection and editing of manuscript. JG contributed to study inception, design, revision of manuscript and approval of final edit.

## Ethical considerations

Not applicable. All patient data are anonymised and data collection was approved by local audit department.
